# Retraction: Dihydroartemisinin suppresses the tumorigenesis and cycle progression of colorectal cancer by targeting CDK1/CCNB1/PLK1 signaling

**DOI:** 10.3389/fonc.2025.1591122

**Published:** 2025-03-25

**Authors:** Frontiers Editorial Office

Following publication, concerns were raised regarding the integrity of the images in the published figures. The authors failed to provide a satisfactory explanation during the investigation, which was conducted in accordance with Frontiers’ policies. As a result, the data and conclusions of the article have been deemed unreliable and the article has been retracted.

This retraction was approved by the Chief Editors of Frontiers in Oncology and the Chief Executive Editor of Frontiers. The authors do not agree to this retraction.

